# Wait, What? What's Going On?— Pregnancy Experiences of Deaf and Hard of Hearing Mothers Who Do Not Sign

**DOI:** 10.1111/birt.12881

**Published:** 2024-10-27

**Authors:** Sanjana Ratakonda, Tiffany L. Panko, Sasha Albert, Lauren D. Smith, Margarita M. Cooley, Monika Mitra, Michael McKee

**Affiliations:** ^1^ Department of Family Medicine University of Michigan Ann Arbor Michigan USA; ^2^ NTID Deaf Health Care and Biomedical Science Hub Rochester Institute of Technology Rochester New York USA; ^3^ Lurie Institute for Disability Policy Brandeis University Waltham Massachusetts USA

**Keywords:** hearing loss, maternal health, perinatal care, qualitative research

## Abstract

**Objective:**

Deaf and hard of hearing (DHH) women experience higher rates of reproductive healthcare barriers and adverse birth outcomes compared to their hearing peers. This study explores the pregnancy experiences of DHH women who do not sign to better understand their barriers and facilitators to optimal perinatal health care.

**Design:**

Qualitative study using thematic analysis.

**Setting:**

Semi‐structured, individual remote, or in‐person interviews in the United States.

**Sample:**

Twenty‐two DHH English speakers (non‐signers) who gave birth in the United States within the past 5 years.

**Methods:**

Semi‐structured interviews explored how DHH women experienced pregnancy and birth, including access to perinatal information and resources, relationships with healthcare providers, communication access, and their involvement with the healthcare system throughout pregnancy. A thematic analysis was conducted.

**Main Outcome Measures:**

The barriers and facilitators related to a positive perinatal care experience among DHH women.

**Results:**

Five key themes emerged. For barriers, healthcare communication breakdowns and loss of patient autonomy highlighted DHH women's struggle with perinatal health care. In contrast, DHH participants outlined the importance of accessible health communication practices and accommodations, use of patient advocacy or self‐advocacy, and assistive technologies for DHH parents for more positive perinatal care experiences.

**Conclusions:**

Perinatal healthcare providers and staff should routinely inquire about ways to ensure an inclusive and accessible healthcare experience for their DHH patients and provide communication accommodations for optimal care. Additionally, healthcare providers should be more aware of the unique parenting needs and resources of their DHH patients.

## Graphical abstract

An obstetrician sits next to a pregnant hard of hearing individual with a hearing aid. There is an exam table nearby.

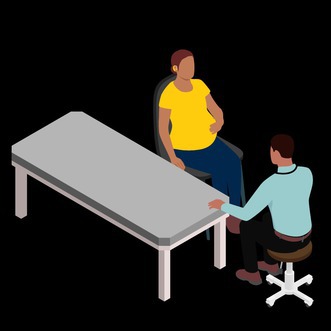

## 1 Background

Almost 5% of women between the ages of 18–39 in the United States report hearing loss [1, 2]. Individuals with hearing loss often face many challenges in accessing health care and report poor physician–patient communication [3]. Effective communication plays a large role during healthcare visits and in patient satisfaction. Hearing loss is associated with adverse health outcomes [4, 5], including higher rates of pregnancy and birth complications [2, 6]. Many deaf and hard of hearing (DHH) individuals experience loss of incidental learning opportunities and language deprivation [7], resulting in health knowledge gaps and low health literacy [8]. DHH individuals are frequently marginalized in health care [9], often due to the nature of hearing loss as an “invisible disability.” Hearing loss is easily overlooked by healthcare providers, especially if hearing aids and/or cochlear implants are not visible or worn. Hearing loss onset also varies widely, with some individuals acquiring hearing loss after they already developed spoken language skills [10]. Even with normal speech, these individuals often struggle to understand their healthcare providers in noisy healthcare environments [11] (e.g., labor and delivery room) or when surgical masks are used [12, 13].

Another challenge arises from the different preferred methods of communication for non‐signers (such as lipreading, captioning, and written notes), with healthcare providers often lacking adequate training on how to meet these communication needs [9]. In addition to these communication challenges and other healthcare barriers due to their hearing status, DHH women, similar to other disabilities, are at higher risk of pregnancy complications (e.g., pre‐eclampsia) and adverse birth outcomes including preterm birth and low birth weight [2, 6, 14, 15]. Despite hearing loss being identified as a risk factor for adverse pregnancy outcomes, there is limited published research specifically focused on pregnancy experiences for non‐signing DHH women. This qualitative study aims to investigate the perinatal healthcare experiences of English‐speaking DHH mothers in the United States who do not use sign language.

## 2 Methods

This study follows Standards for Reporting Qualitative Study [16] As part of a larger study on the pregnancy experiences and outcomes of DHH women [17, 18, 19], interviews with 22 DHH English speakers (non‐signers) were conducted, and a sample size was chosen based on data saturation [20]. Data collection stopped when no new themes emerged from the data. Eligible women were between 21 and 50 years of age and gave birth within the past 5 years in the United States. Participants were recruited by a mix of purposive, convenience, and snowball sampling via email, social media, and in‐person recruitment at community events. To maximize representation, study flyers and social media graphics were distributed widely, targeting organizations serving DHH people, including those from different racial and ethnic minority groups. Participants were asked about their access to pregnancy information and resources, communication access, satisfaction with healthcare providers, and experiences with the healthcare system throughout pregnancy. When participants reported multiple pregnancies, they were invited to discuss the pregnancy they perceived as most relevant. The Perinatal Health Framework for Women with Physical Disabilities [21] was adapted for use with DHH women. This framework served as a guide to our investigator team due to its focus on the needs and barriers that are commonly faced by pregnant disabled women, including DHH women (e.g., disability stigma, healthcare barriers, and higher risk for adverse outcomes). The framework use allowed the development of a much‐needed construct to better understand both specific and general relationships between perinatal health and health care and hearing loss. Ethical approval was obtained from the authors' Institutional Review Boards. This paper focused on the subset of women who self‐identified as DHH* and preferred to communicate in spoken English (non‐signers) as their primary language. Findings from interviews with Deaf American Sign Language (ASL) users were published elsewhere [18].

### 2.1 Data Collection

From May 2018 to November 2019, the research team conducted 90‐min‐long semi‐structured qualitative interviews with 22 DHH participants. Initially, we conducted in‐person interviews at three main sites (Rochester, NY, *n* = 3; Chicago, IL, *n* = 2) with interviewers experienced working with DHH individuals. To increase participation and diversity, we augmented our approach through a national recruitment strategy and remote interviews; interviews not conducted in‐person were conducted real time via the online BlueJeans video conferencing app with available chat support (e.g., to clarify any misunderstood words) for the remaining 17 participants. Prospective participants that did not have in‐person interviews completed an online Qualtrics questionnaire to determine their eligibility. Eligible participants were directed to a web‐based informed consent and a demographic questionnaire before being scheduled for interviews. Participants received a $50 gift card. Interviews were recorded for professional transcription.

### 2.2 Positionality and Qualitative Research Expertise

The team (three people of color, two DHH, two Latine, and one interpreter) led the interviews and coding work. Six of the team members have prior experiences with conducting qualitative studies with DHH individuals.

### 2.3 Analysis

Members of the research team reviewed transcripts to identify initial set of themes, helping to develop a codebook with preliminary codes. The codebook was updated, as transcripts were revisited to refine the coding scheme as additional themes emerged. The coding team conferred throughout this process to finalize a codebook containing descriptors that encompassed the participants' pregnancy experiences within the healthcare system. Three coders coded all the transcripts and met regularly with the lead investigator to review and clarify codes. Given the primary aim of the study was to delve into participants' experiences, rather than generating a theory, a descriptive qualitative approach as described by Sandelowski [22, 23], was employed. In the descriptive qualitative approach, the data collection and analysis focused on staying close to the participants' experiences and related quotes, without over‐abstracting during analysis. The coding team included researchers who identified as DHH, and was completed with Dedoose Version 9.0.90 [24] using the team's established codebook. All coding disagreements were resolved through consensus. Recurring themes were developed under the domains of barriers and facilitators using thematic analysis [25, 26, 27].

## 3 Results

**FIGURE 1:**
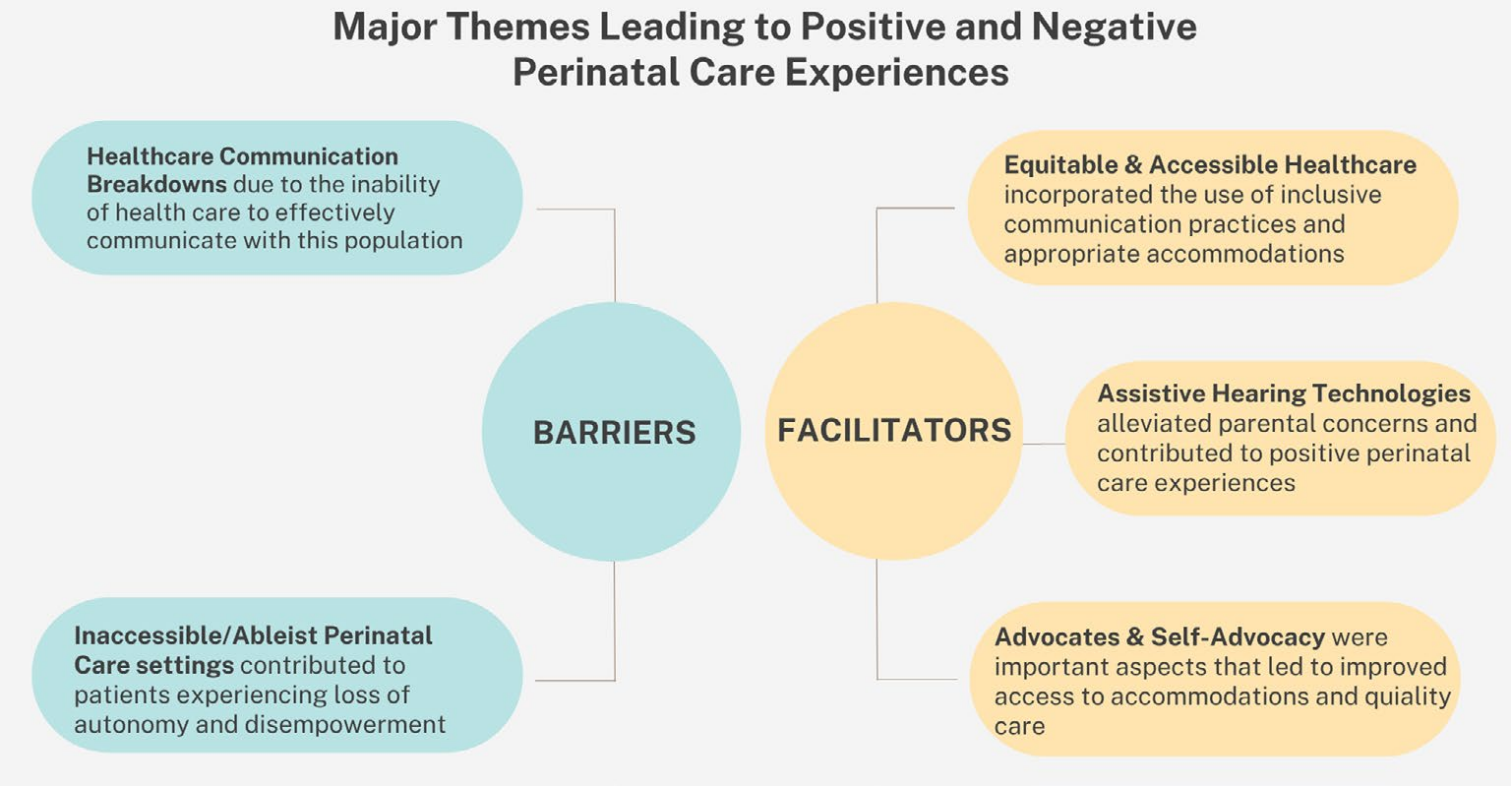
Major themes leading to positive and negative perinatal care. [Colour figure can be viewed at wileyonlinelibrary.com]

The 22 participants were predominantly white, married, reported an annual income less than $80,000 and reported a 4 year college degree or higher. Fifteen participants had prelingual hearing loss. Most participants with multiple pregnancies selected their first pregnancy to discuss during the interviews (Table 1). The key themes outlined below include both the barriers and facilitators to quality perinatal care experience (Figure 1).

Barrier Theme 1 Healthcare communication breakdowns due to the inability of healthcare to effectively communication with this population.

**TABLE 1:** Participant characteristics (*n* = 22).

	*N* (%)
Age, years	
Mean (range)	32.7 (21–44)
Race	
White	18 (81.8%)
Black or African American; Asian; American Indian or Alaska Native	2 (9.1%)
Bi‐racial/Other	2 (9.1%)
Hispanic/Latina	1 (4.5%)
Household income	
Less than $80,000	11 (50.0%)
More than $80,000	11 (50.0%)
Age of Hearing Loss Onset	
Prelingual	15 (68.2%)
Postlingual	6 (27.3%)
Don't Know	1 (4.5%)
Highest level of education	
2‐year college degree or less	8 (36.4%)
4‐year college degree	2 (9.1%)
Graduate degree	12 (54.5%)
Marital status	
Single	7 (31.8%)
Married	15 (68.2%)
Family size	
One child	11 (50.0%)
Two or more children	11 (50.0%)
Birth Discussed	
1st child	12 (54.5%)
2nd child	8 (36.4%)
3rd or later child	2 (9.1%)
Delivery type	
Vaginal	15 (68.2%)
Cesarean Section, planned	1 (4.5%)
Cesarean Section, emergency	6 (27.3%)

All participants reported some level of communication difficulties as a result of the healthcare system's inability to accommodate their hearing loss. They reported feeling unsure, confused, and at times, disempowered. Perinatal care settings, especially birthing and hospital locations, were challenging communication environments due to the use of surgical masks, quickly changing situations (e.g., concerning fetal monitoring), complex terminology, lack of healthcare team members' awareness or training on how to care for DHH women, and no notifications to alert healthcare providers and staff about a patient's hearing loss. One participant reported that communication barriers resulted in a tense and marginalizing birthing experience.

… [nurses would] be talking to the computer, and so, “How's your pain level today?”… I'm like over here, and “I can't hear you. You have to look at me when you talk”… And when the labor, and the pains were happening, I didn't hear anything… the frustrating part was they were saying things, but I couldn't hear them, and I would kind of have to be like, “What? What's happening?” “The baby's heartbeat is dropping.”… I don't think I've ever told my husband this, but my husband actually recorded, like the last 15 minutes of labor…, and when she finally came out, you could hear me multiple times kind of ask, “What is it? What is it?”

Participants found that the perinatal healthcare settings were not designed to effectively care for them—and when attempts were made to accommodate DHH patients, they often were inappropriately tailored. One participant reported that an antenatal emergency department visit was delayed because the hospital was locating a sign language interpreter even though she did not sign. Another felt that accommodations, if available, were limited to sign language interpreter or note writing. Furthermore, many perinatal healthcare providers relied on telephones to connect with patients outside of the clinic. This placed DHH women at a disadvantage as many felt unable to use the telephone, and several participants reported using their spouses or family members to listen to their voicemails or calls.

Through phone calls,… [his nurses] would leave messages to me, which I think is probably not the smartest way for me, because especially, if I pick up the phone and have a dialogue with them, I'm working so hard to listen, and hear what they're saying that I'm not actually, like, comprehending – remembering, like wait a minute, what are my levels? And what's that word you said?… So, I have my husband listen to it with me. So, oh, here's my levels, and they say I have this condition, preeclampsia, whatever it is… when you're deaf/hard of hearing, you're just struggling to just get by, and maybe if I think about it more, like okay, can you please communicate to me through e‐mail?

Barrier Theme 2 Inaccessible/Ableist perinatal care settings contribute to patients experiencing loss of autonomy and disempowerment.

Six participants reported requiring an emergency C‐section with three expressing difficulty understanding their care providers during this critical period. Consequently, they found themselves uninformed and under pressure to hastily sign surgical consent forms.

I didn't realize that we were building up to a C‐section… Then the doctor comes in like, ‘Well, time for a C‐section.’ I'm like, ‘Wait, what? What's going on?’ I'm like, ‘What? Why? I just need more data’… My nurse was just going with it. She's like, ‘Yes, alright let's get ready for this.’ I'm like, ‘Oh my God,’ just a lot of shocking, kind of moving fast once it started happening. It wasn't quite an emergency C‐section… If they had told me hours ahead of time that this was on the radar, like, ‘Let's start thinking about this’ and ‘What are our options?’ that would have been helpful. But there was really nobody [I could understand]. I don't think anyone realized I didn't hear all of that.

Another reported experiencing a similar loss of patient autonomy in which she was unable to understand healthcare team members behind surgical masks as she underwent a surgical procedure shortly after the recent death of her infant.

I had to go through a surgical procedure after losing [my baby]. No one explained to me what that was like and I brought my husband with me. I brought him… because I can't understand them when they have masks on. I can understand [husband] but they wouldn't let him be in the [operating] room… so that was very stressful.

Sixteen participants relied significantly on their social networks to facilitate communication with their healthcare providers. The use of family and significant others helped minimize missing out on important health information. One participant would not go to her prenatal appointments without her hearing boyfriend because she felt too vulnerable to adequately self‐advocate for accommodations. In some cases, the healthcare team sought hearing family members or significant others to serve as proxy voice interpreters or to help with accommodations. Another participant reported that her husband was involved with the birthing process while simultaneously assisting with communication. Additionally, two participants expressed disempowerment, which led to a loss of patient autonomy, when staff members spoke to their partners instead of directly to them:

My boyfriend was there [after delivery], so he was able to help me. It felt like my boyfriend took over… the nurses, they realized he's hearing, so they just talked to him, not me. Hello, I'm the mother! I'm right here!

Another participant reported experiencing audism (“belief that the ability to hear makes one superior to those with hearing loss”), when she was notified about her baby's normal hearing assessment:

There was one thing that irritated me was when my baby took the hearing test and he passed, they came up to us and told us, ‘Great news, he's hearing.’… I was a little conflicted. She should have said… the baby passed the test. Not ‘Oh, great news, he's not deaf.’ It made me feel like I should feel lousy that I'm deaf.

Similarly, all participants shared key communication facilitators and recommendations on how to improve communication in perinatal care settings.

Facilitator Theme 1 Equitable & accessible health care incorporates the use of inclusive communication practices and appropriate accommodations.

Participants highlighted the importance of accessible communication practices, standardized data collection that includes disability types and accommodation needs, and increased hearing loss awareness. Participants regarded these steps as critical to ensuring an accessible, inclusive, and positive perinatal experience. When the appropriate accommodations were provided, participants reported positive healthcare experiences. One participant explained how her healthcare team adapted their communication techniques to better align with her needs as a DHH patient:

[The physician] looked directly at me, [with her] undivided attention. [The caregiving team] spoke loud and clear enough for me, and [they were] at an angle so that my husband also could see, we could both see. I think [they] really made sure that they were clear and loud and understanding and slow enough for us to follow along.

Multiple participants actively sought healthcare providers who were willing to learn to effectively communicate or already incorporated successful communication practices. One participant highlighted how effective communication led her to continue with the same physician for her perinatal care:

When I went to the first appointment [the physician] was great,… she was very clear… She understood that I'm hard of hearing [and] would never say anything without looking at me first. [The physician] always talked to me, meaning if one of my parents or if a sibling came with me, she would always talk [directly] to me… So, I really appreciated that. and that's why I stuck with her.

This participant felt comfortable with her care team due to their willingness to work with her to ensure that communication was accessible. This resulted in continuity with this provider. Additionally, participants valued cultural humility. One participant expressed gratitude for her healthcare provider for being upfront about his lack of experience working with DHH women yet a willingness to learn.

[My physician] was very accommodating. He said he hadn't had experience with [a cochlear implant] but just to let him know what [is needed] so he was very accommodating.

Effective communication and support from the healthcare team were reported by participants as being critical for participants to receive high‐quality perinatal care. This required notification of healthcare providers and staff of the participants' hearing loss and accommodation needs. One participant recalled how her care team worked together to ensure her needs were being met:

[The provider] was wonderful about it. She just said that any time you needed something to let her or the nurses know and then they would do the best to let everybody at the hospital know, too, and they put it in my chart.

Another participant reported that charting her hearing loss in the medical record helped to improve communication access and quality. “The doctor knew I have a history of hearing loss. It is basically all in… [chart]. They knew not to wear masks. If they did they would take it off to talk and then put it back on.”

Facilitator Theme 2 Advocates and self‐advocacy are important aspects leading to improved access to accommodations and quality care.

Self‐advocacy was a recurring theme across participants. Self‐advocacy was important to both inform healthcare providers about their communication needs and to receive appropriate accommodations and support. One participant described how she learned to advocate for herself:

… I tell people right away so that there's no confusion. In the past, I used to leave it up to other people to guess or to provide any resources I may need,…, I found that I need to make clear that this is my situation. I need you to maybe remove your mask so I can hear you. I need to tell people what I need from them to make communication easier”. In addition, many participants noted that they had support from family during their visits. Another participant recalled having her husband support and advocate for her during times she was unable to do so for herself: “My husband was there with me, and he is a very good advocate for me if people aren't [speaking clearly and directly at me] and I wasn't able to do that while in labor.

Other participants felt that patient advocates should be non‐family members to ensure impartiality. One participant commented that a doula was an invaluable advocate for her communication needs.

And when I was interviewing doulas, I made sure I found someone that I felt comfortable with, that I could understand, that they understood and respected that I was hard of hearing. So, they were very good about how to communicate with me. And they would advocate on my behalf if any issue came up. Sometimes you know, when you're pregnant, [the doula] would repeat things that the nurse said or if I wasn't sure. Or [the doula] would remind the anesthesiologist – she's hard of hearing, you need to look at her… That's why I hired her, to have her there, to be there for me.

Facilitator Theme 3 Assistive hearing technologies alleviate parental concerns and contribute to positive perinatal care experiences.

Participants expressed concerns about their inability to hear their baby cry or make noises, especially during sleep. Some participants reported sleeping with their hearing aids and/or cochlear implants or sleeping on the side of their non‐hearing ear:

At the time, I didn't know what was really out there for options for hard of hearing or deaf, and now… I see there are different vibrating alarms, basically like a vibrating alarm clock, but for a baby monitor, flashing lights, that type of thing. [Unfortunately] I slept on my deaf side, so I could hear what was going on, because my husband sleeps through everything.

However, participants found that DHH parenting resources could significantly improve the quality of the perinatal experience. A participant reported using various technologies with non‐auditory alerts to help monitor her infant and allow her to sleep.

Before I was pregnant, I actually did not sleep with my hearing aids. I wear a hearing aid and a cochlear implant. So, after he was born, I always sleep with now a cochlear implant on to make sure I can hear him crying, even though the bassinette is right next to me… So, that was a change for me because I'm not used to sleeping with the aids on. But, you know, I was never fully asleep because I would always be on the alert for a cry or movement or something. [Now] I have a video monitor there… and you can set it up to get any kind of notification sent to your phone, so my phone will vibrate if there's a movement, if there's a sound, whatever you prefer… So, I'm doubling up on everything to make sure I can hear him when he needs me.

## 4 Discussion

This study found that non‐signing DHH women faced significant barriers that adversely affected the quality of their perinatal healthcare experiences, resulting in loss of patient autonomy, including instances of inadequate informed consent for surgical procedures. Despite the relatively common occurrence of hearing loss, most perinatal healthcare systems are ill‐equipped to deliver inclusive and accessible perinatal care for signing and non‐signing DHH women. One contributing factor is the inconsistent documentation and adherence of communication preferences and accommodations for DHH patients [9].

Additionally, healthcare providers and staff often lack training on how to effectively identify and care for DHH women. The Americans with Disabilities Act of 1990 (ADA) and Section 1557 of the Affordable Care Act (ACA) mandate that public and private healthcare facilities provide equal healthcare access to services for people with disabilities, including DHH women. Section 4302 of the ACA and a recent Biden Executive Order on Advancing Racial Equity and Support for Underserved Communities Through the Federal Government both require data collection standards for inclusion in health surveys, including those with disabilities [28]. Section 5307 of the ACA authorizes federal funding to train healthcare professionals in disability competent care, including hearing loss.

Despite these legal requirements that advocate for disability health equity, the documentation of communication preferences for people with hearing loss and the provision of necessary accommodations for DHH individuals in healthcare settings remains poor [9, 29, 30, 31]. Additionally, studies have indicated that healthcare providers may hold biases and negative perceptions (e.g. audism) about DHH patients [9, 31, 32, 33, 34]. Audism, along with hearing loss stigma, are related to the negative effects of ageism and the harmful connotations given to those with disabilities [34]. These harmful attitudes toward DHH patients combined with insufficient clinical training in best practices for caring for this patient population lead to suboptimal healthcare delivery, mistrust, and patient disempowerment [6, 9, 30]. When healthcare providers and staff prioritized communication accessibility and inclusive environments, participants reported positive and affirming perinatal experiences. Even when healthcare providers lacked training on effectively communicating with DHH patients, participants valued providers' demonstration of cultural humility and willingness to learn. Participants highlighted the importance of documenting their hearing loss on patient boards, electronic medical records, healthcare team discussions, and birth plans. The Hearing Loss Association of America provides a communication access plan [35] that can be easily adopted into birth plans for DHH women to inform the healthcare team on specific health communication needs. Other key findings centered on the importance of advocacy, either through self‐advocacy or the involvement of patient advocates, to enhance accessibility and support. While most participants relied on family members or partners to assist them with advocating for accessible communication or even to serve as proxy interpreters, two participants interestingly opted for doulas to assist with this. Doulas can offer a more impartial approach concentrating on supporting the DHH women and their communication needs, freeing up significant others to witness the birth of a child or provide emotional support to the DHH mother. Additionally, self‐advocacy often evolved through negative experiences with earlier pregnancies rather than through a formal training program.

### 4.1 Strengths and Limitations

This study has several limitations. The sample was largely highly educated, which may be associated with higher level of self‐advocacy and health literacy, and consequently more positive birth experiences. This may result in an underrepresentation of adverse experiences of more marginalized and less educated DHH women. Additionally, DHH women of color were underrepresented, underscoring the need for caution in generalizing the study's outcomes to a more representative population of non‐signing DHH women. While our data collection relied on participant self‐reporting, it is important to acknowledge the potential presence of recall bias. However, participants were able to provide detailed narratives suggesting a high likelihood of accurate recall. Despite these limitations, this is the first perinatal care study to conduct qualitative interviews with DHH English speaking women, a group not well understood, in contrast to Deaf women who communicate in ASL.

## 5 Conclusions

The communication needs of DHH non‐signing women are often overlooked, and it is important to acknowledge their diverse communication preferences and needs. The findings from this study highlight the need for incorporating accommodations for DHH patients and identification of communication approaches into obstetric intake processes. DHH women are at high risk for adverse perinatal outcomes and experiences. Training is needed to inform providers and staff on inclusive and accessible communication practices, provide relevant tailored perinatal and parenting resources for DHH women, and reduce existing health inequities.

## Ethics Statement

The University of Michigan Institutional Review Board approved this study in January 2019 under HUM00137149.

## Conflicts of Interest

The authors declare no conflicts of interest.

## Data Availability

To protect participant confidentiality, data sharing is not possible.

## Endnote

* Deaf and hard of hearing (DHH) is used to define those who identify as having a hearing loss, regardless of their hearing loss severity or their communication preference.
